# A Rare Case of Gunshot Wound, with Recovery

**Published:** 1886-06

**Authors:** C. K. Gregg

**Affiliations:** Ramos Arispe, Mexico


### A RARE CASE OF A GUN SHOT WOUND WITH RECOVERY.

*By C. K. Gregg, M. D., Ramos Arispe, Mexico.*

(For Daniel's Texas Medical Journal.)

PHILLIPE DELBOSQUE, aet. 21, whilst imbibing too freely of a favorite Mexican liquor, "Pulque," at a "Pulqueria," on Sunday afternoon April 28th, attempted to draw his pistol, a 45 cal. S. & W., (secreted under his vest, and between his pants and shirt), and which was accidentally discharged.

He was removed to his home at once, and seen a few moments after the accident. I found that the bullet had entered the hypogastric region of the abdomen, to the right of and above the bladder, taking a downward and oblique course, and making its exit from the body at the apex of left testicle, producing a slight wound on the inner side of the left thigh, and lodging in his drawers. As he had passed his water shortly before my arrival, and which was free from blood, (the bladder having been quite distended prior to accident.) I came to thc conclusion, therefore, that the bullet must have passed above and near the neck of the bladder, without injuring that important viscus, under the symphisis pubis and following the spermatic cord made its exit as above stated. Hemorrhage was slight, but shock very great; however, as apparently the bladder had not been injured, I gave a favorable prognosis. Fearing subsequent peritoneal trouble, the bowels were ordered to remain at rest, and all purgative interference strictly prohibited. He was given a hypod. of morphia, and cold applications applied to both wounds ; liquid diet, and absolute rest enjoined, for fear of secondary hemorrhage.

For the first few days he did exceedingly well, with the exception of suffering some pain, which however was easily relieved by hypods. of morphia. On the third day he had some fever, temperature reaching 101 degrees Fahrenheit, (highest point reached.)

On the fifth day suppuration commenced from the lower wound, and on the same day pain in testicle and abdomen was excruciating. Scrotum, penis and left iliac region of abdomen became ecchymosed and remained so until convalescence was well established. Suppuration, especially from the testicular wound was very profuse, lasting nearly two weeks.

No symptoms of peritonitis manifested themselves, which I attributed in a measure to the quietude of the bowels for the first eight days. He always passes his water freely except when under the influence of morphine. About the eighth day he was greatly annoyed by nausea and gastric irritation, which however, was easily relieved by an effervescent mixture of pot. bicarb and acid tartaric. When cold applications were no longer necessary, the remainder of the treatment consisted in applying iodoform and zinc ointment alternately over the wounds which caused them to heal very quickly.

In less than one month my patient was able to ride on horseback and attend to his duties as "*Gendarma.*"

